# Experiences of a long-term randomized controlled prevention trial in a maiden environment: Estonian Postmenopausal Hormone Therapy trial

**DOI:** 10.1186/1471-2288-8-51

**Published:** 2008-08-01

**Authors:** Sirpa-Liisa Hovi, Piret Veerus, Mati Rahu, Elina Hemminki

**Affiliations:** 1 Finnish Office for Health Technology Assessment (Finohta), National Research and Development Centre for Welfare and Health (STAKES), PO. Box 220, FI-00531 Helsinki, Finland; 2 National Institute for Health Development (TAI), Department of Epidemiology and Biostatistics, Hiiu 42, 11619 Tallinn, Estonia; 3 Estonian Centre of Excellence in Behavioural and Health Sciences, University of Tartu, Department of Psychology, Tiigi 78, 50410 Tartu, Estonia; 4 Health and Social Services, National Research and Development Centre for Welfare and Health (STAKES), PO. Box 220, FI-00531 Helsinki, Finland

## Abstract

**Background:**

Preventive drugs require long-term trials to show their effectiveness or harms and often a lot of changes occur during post-marketing studies. The purpose of this article is to describe the research process in a long-term randomized controlled trial and discuss the impact and consequences of changes in the research environment.

**Methods:**

The Estonian Postmenopausal Hormone Therapy trial (EPHT), originally planned to continue for five years, was planned in co-operation with the Women's International Study of Long-Duration Oestrogen after Menopause (WISDOM) in the UK. In addition to health outcomes, EPHT was specifically designed to study the impact of postmenopausal hormone therapy (HT) on health services utilization.

**Results:**

After EPHT recruited in 1999–2001 the Women's Health Initiative (WHI) in the USA decided to stop the estrogen-progestin trial after a mean of 5.2 years in July 2002 because of increased risk of breast cancer and later in 2004 the estrogen-only trial because HT increased the risk of stroke, decreased the risk of hip fracture, and did not affect coronary heart disease incidence. WISDOM was halted in autumn 2002. These decisions had a major influence on EPHT.

**Conclusion:**

Changes in Estonian society challenged EPHT to find a balance between the needs of achieving responses to the trial aims with a limited budget and simultaneously maintaining the safety of trial participants. Flexibility was the main key for success. Rapid changes are not limited only to transiting societies but are true also in developed countries and the risk must be included in planning all long-term trials.

The role of ethical and data monitoring committees in situations with emerging new data from other studies needs specification. Longer funding for preventive trials and more flexibility in budgeting are mandatory. Who should prove the effectiveness of an (old) drug for a new preventive indication? In preventive drug trials companies may donate drugs but they take a financial risk, especially with licensed drugs. Public funding is crucial to avoid commercial biases. Legislation to share the costs of large post-marketing trials as well as regulation of manufacturer's participation is needed. [ISRCTN35338757]

## Background

### The importance of the trial process

Preventive medications require long-term trials to show their effectiveness and harms. Carrying out a trial of long duration is demanding for the researchers. Very few reports have described the long-term trial process in practice [1,2]. When trial processes are not described publicly, useful knowledge fails to accumulate. Especially for trials that are unable to meet their targets, information about the process is important to aid other researchers in anticipating and avoiding similar problems. Also, trials that have successfully coped with unanticipated difficulties should report their success stories.

Previous studies of the process of preventive trials have mainly concentrated on the recruitment process [1,3-5], failures in recruitment [6], the non-medical intervention effect on compliance [7,8], and randomization [9]. Oakley et al. [1,2] have reported a trial process on social support in motherhood and on peer-led sex education. They found that an evaluation of the process was integral to understand the outcomes. In a trial on the delivery of very low birth weight infants, Lumley et al. [9] failed to achieve randomization because of a critical shift in obstetric practice. In Finland the pilot for a non-blind, patient-managed trial on hormone therapy (HT) revealed several obstacles to the main trial, including the difficulty to discontinue HT and a negative attitude among Finnish physicians towards the trial [5].

We have found no process description of a successful trial involving preventive drug therapy. To run a trial over many years involves a significant risk that obstacles will emerge. The purpose of this article is to describe the research process in a long-term randomized controlled trial, the Estonian Postmenopausal Hormone Therapy trial (EPHT), and to discuss the impact and consequences of changes in the research environment. We hope that this report of the EPHT trial process would provide researchers with valuable information for planning and carrying out long-term trials.

## Methods

### The EPHT trial

The EPHT trial aimed to study the impact of postmenopausal hormone therapy (HT) on diseases, subjective well-being, social effects, health service utilization and health care costs. Furthermore, we aimed to study the impact of blinding on trial process and outcomes. In regard to the long-term health effects, the aim was to study whether HT increases the risk of cancers, and decreases the risk of heart and cardiovascular diseases, fractures and metabolic diseases. Because the EPHT alone did not have power enough to detect HT effects on diseases, these outcomes were planned to be pooled with the Women's International Study of Long-Duration Oestrogen after Menopause (WISDOM) to increase its power. The independent aims of the EPHT trial were to study whether HT increases women's well-being and decreases the prevalence of their symptoms, how HT affects experience of the climacteric and ageing, and partner relationship. The EPHT was specifically designed to investigate whether HT increases health services utilization and therefore the non-blind sub-trial was needed, which also made possible to study methodological questions like the effect of blinding on recruitment and trial results.

A pilot showed that doing a trial on HT in Finland was unlikely to succeed due to women's and physicians' strong preferences to HT [5], and it was decided to carry out the trial (EPHT trial) in Estonia. Originally we had planned to do our trial in close co-operation with WISDOM. In the mid-1990s, the UK-based WISDOM unsuccessfully sought financing from the European Union (EU) for a large European HT study [10]. The UK Medical Research Council (MRC) decided to finance the trial covering the UK, Australia, and New Zealand. In view of this decision, we planned our trial as an independent study, though still working closely with WISDOM.

**Figure 1:**
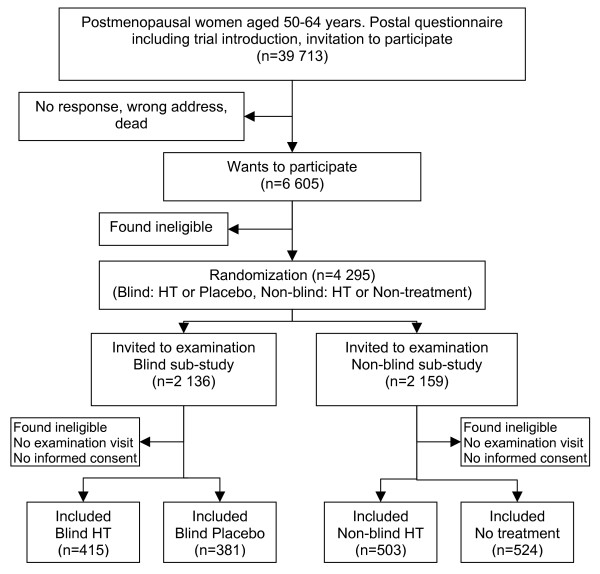
Recruitment flow.

The planning of the trial was started in 1995. It was a four-arm randomized controlled preventive trial on HT, originally planned for five years exposure, consisting of a blind and a non-blind sub-study (Figure 1). The blind sub-study represents a traditional randomized, double-blind, placebo-controlled trial except for the early randomization prior to informed consent, whereas, the non-blind sub-study was a randomized controlled trial with an open-label HT arm and a non-treatment arm. For details of the trial design and methods, see [11].

Participants were recruited using a postal questionnaire to establish eligibility and to ask whether they wished to participate in the trial. Those positive and potentially eligible women were randomized to the four trial arms. Women were mailed a letter briefly describing either the blind or non-blind sub-study to which they had been randomized and inviting them to the recruitment examination in one of the study clinics. [12] The EPHT trial had three study centers, two women's clinics in Tallinn, the capital of Estonia, and a university women's clinic in Tartu, southern Estonia. Trial staff in each study centre consisted of 2–3 gynaecologists and two midwives in each clinic, 13 altogether.

Local coordination in Estonia was at the Institute of Experimental and Clinical Medicine (EKMI, later the National Institute for Health Development, TAI). In 1998 the trial received a positive statement from the Tallinn Committee of Medical Ethics, and the trial was registered at the State Agency of Medicines in the same year. By agreement drugs were to be donated by Wyeth, a US-based pharmaceutical company, to the EPHT trial via the WISDOM trial in the UK.

The trial drug was licensed in the USA and was a combined regimen of conjugated equine oestrogens 0.625 mg (CEE) and medroxyprogesterone acetate 2.5 mg (MPA). A similar drug with a higher MPA dose (10 mg) had already been licensed in Estonia. Women were to visit the study clinics semi-annually to collect their trial medication, and annually for an examination by the study physician; this did not apply to women in the no-treatment-arm in the non-blind sub-study, who were to visit only if needed.

Financing for the trial came from public sources such as the Academy of Finland, STAKES, the Finnish Ministry of Education, and the Estonian Ministry of Education and Science, while the Universities of Tartu (Estonia) and Tampere (Finland) and the Estonian National Institute for Health Development (TAI, previously EKMI) offered institutional support.

## Results

### Impact of other studies on EPHT

**Figure 2:**
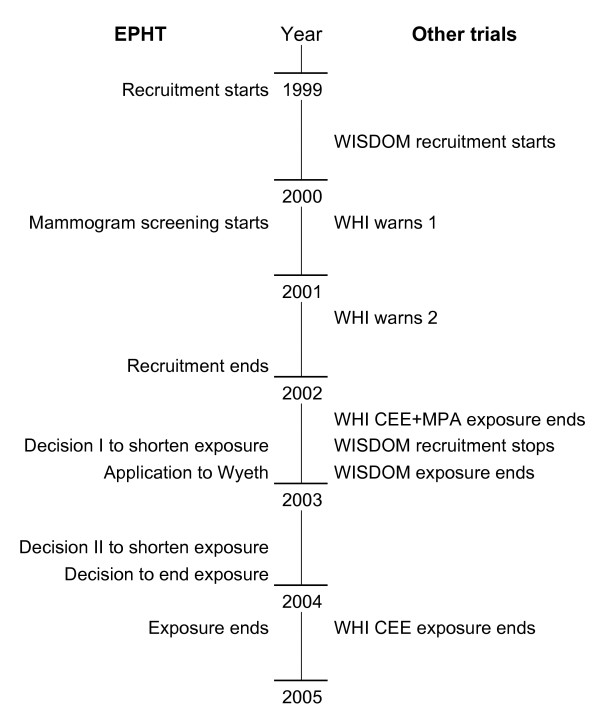
Time flow of the trial.

Recruiting occurred from 1999–2001 (Figure 2) and 1823 women joined up. Half a year after recruiting was completed (July 2002), the Women's Health Initiative (WHI) prematurely published its results in the USA. This had a big influence on our trial, both directly and through its impact on WISDOM. Originally we had calculated that our trial exposure would have been completed before the WHI is ready. The WHI was initiated in 1992 with a planned completion date of 2007. It included two large trials to investigate the effects of HT on the morbidity and mortality of postmenopausal women aged 50 to 79. Between 1993 and 1998, the WHI randomized 16 608 women with a uterus to the estrogen plus progestin trial [13] and 10 739 women without uterus to the estrogen only trial [14] to be treated for an average follow-up of 8.5 years [13].

Already in 2000 and 2001, the WHI Data and Safety Monitoring Board recommended that the participating women should be informed that the original hypothesis of cardiovascular protection was no longer likely, but that the trial would continue because the balance of risks and benefits remained uncertain. In July 2002, the WHI estrogen-progestin trial was stopped because the breast cancer risk comparison exceeded the pre-defined limits and the overall risks were seen to exceed the benefits as measured by the global disease index [13]. By summer 2003, results in several articles emerging from the WHI trial showed that HT is not safe for disease prevention [13,15-17]. The trial with estrogen alone continued until early 2004 when the intervention was withdrawn because the original hypothesis of estrogen preventing the risk of cardiovascular diseases was unlikely [14].

The first time we became aware of the WHI warnings to its trial participants was in 2001. The Trial Steering Committee kept itself updated via the WHI web site where we learnt that a warning had been given also in 2000. We tried to get further information with direct contacts, but were not successful. We had no reason to send further information to the EPHT trial women in 2001, because the original written information at recruitment had stated that "HT probably decreases cardiovascular diseases (CVD), and estrogen is thought to have specific effects on blood coagulation and plasma lipid concentration, but their effect on CVD is still not clear. It is assumed that HT decreases the risk of myocardial infarction, but it may increase the risk of thrombosis for some women."

The EPHT Trial Steering Committee had its meeting in September 2002 and unanimously decided to continue the trial unless WISDOM discontinued for ethical reasons. During the discussion it was suggested to open the blindness in the blind sub-study and continue EPHT as a totally non-blind trial, but that was not supported. We were satisfied with the way the recruitment letter described the uncertainties of HT effects. Thus, we did not change the protocol in 2002.

By autumn 2002, WISDOM had recruited 5 700 women. After WHI prematurely stopped its first trial in July 2002, WISDOM's Data Monitoring and Ethics Committee recommended WISDOM to continue as long as women were informed of the current state of knowledge. Likewise, the Trial Steering Committee recommended continuation as they found no strong scientific or ethical reasons to stop. However, the Medical Research Council (MRC), who were the main funding agency, decided to convene an Independent International Committee to review the WHI findings, the progress of WISDOM and other evidence. The Committee concluded that "WISDOM was unlikely to provide substantial evidence to influence clinical practice in the next 10 years" [10]. MRC decided in October 2002 to stop the funding of WISDOM on the basis of the lack of importance [18].

The halting of WISDOM in October 2002 did not have an immediate effect on the continuation of our trial. In November 2002 the EPHT Data Monitoring Committee (DMC) made the annual interim analysis of the data and found no results that would have demanded cessation of the trial. A strong argument for us to continue the exposure was the WISDOM Steering Committee's recommendation to continue WISDOM. An important reason behind the MRC decision to stop WISDOM was financial rather than safety concerns. We wanted to obtain data to answer our research questions other than effects on diseases. However, as the WHI results suggested that breast cancer risk increased by the length of exposure, the EPHT Trial Steering Committee decided to shorten the trial treatment to four years from the original five for those women who had not yet been in the study for 4 years (December 2002).

We kept the trial physicians and midwives informed about all new results in other trials via information letters and personal discussions. Also, the Tallinn Medical Research Ethics Committee was regularly informed with updated information from other HT trials. The participating women were kept up to date on the results of other trials and their influence on EPHT as well as with the process of the EPHT trial with an annual newsletter in Estonian. In September 2002 women were told why WHI estrogen-progestin trial was stopped, and were given the disease outcomes per 10 000 women for those using or not using HT. Women were told that they had been in the trial for a shorter period than women in the WHI, and women with less than four years of exposure were encouraged to continue the trial treatment. We encouraged the women to contact the researchers if they wanted more information.

In spite of all the given information no decrease in adherence was detected, and only a few women had contacted the trial staff because of the new warnings. The media coverage of the 2002 results of the WHI estrogen-progestin trial was very low and did not raise any public discussion in Estonia. Instead, in Finland the WHI results were widely discussed both in the professional and lay press [19]. EPHT investigators in Finland were interviewed and they explained that the WHI results should result in decreasing long-term HT use. Many leading Finnish gynaecologists belittled the significance of the WHI results with various arguments [19].

In August 2003, following the release of the WHI results of HT effects on dementia, cognitive functions, and quality of life [15,17,20], and the results from the Million Women Study on HT effects on breast cancer [21], the Trial Steering Committee shortened the exposure in our trial for the second time. The exposure was shortened to three years for women who had by that time received it for less than three years.

In December 2003, our trial DMC had its annual meeting where all cumulative information about other HT studies were presented, as well as the results of the interim analysis of the EPHT data. EPHT data showed an unfavourable effect of HT on CVD, but not statistically significant. The DMC had no pre-defined rules on how to interpret data from other trials, but decided to recommend ceasing the trial treatment, as results from other trials were against the preventive use of HT. Based on the DMC recommendations, the Trial Steering Committee stopped the treatment over a period up until May 31^st ^2004 to enable a final medical examination to all women. As a result, 597 women received trial treatment at least for four full years, 808 for three years, and the rest 418 at least for two years (Table 1).

**Table 1:** Numbers of women by the length of exposure (years) at the time of stopping exposure in the EPHT trial (May 2004).

Sub-study	Arm	Length of exposure (years)			
			
		2–2.9	3–3.9	4+	Total
**Blind**	Hormone therapy	89	198	128	415
	Placebo	85	182	114	381
**Non-blind**	Open-label hormone therapy	127	212	164	503
	Control	117	216	191	524

Total		418	808	597	1823

Our trial received its drugs from Wyeth via WISDOM. After WISDOM was discontinued, we sent Wyeth an application for registration in their trial registry to receive more drugs. However, the registration was never finalized: during the lengthy negotiations, we had twice shortened the trial, and in the end no more drugs were needed.

Besides abbreviating the trial duration, the new information coming out from other trials led to a lot of additional work: we had to thoroughly analyze the data and consider its impact on our study protocol, to inform both women and the clinical staff, as well as to monitor news reports.

### Changes in research environment

Between the initial planning year (1995) and the halting of the intervention (2004), Estonian society changed very rapidly. Estonia had been a part of the Soviet Union with a planned economy up until 1991, when the independent Estonia had adopted a liberal market economy. By 1994–95 pharmacies had been privatized and the availability of drugs was no longer a problem and medicine choices were determined mostly by prescribers [22]. In 2004 Estonia became a member of the European Union and its economic and scientific contacts with Western Europe increased and income had increased, but less for poor people [23]. A better financial situation and availability of HT offered women in the blind sub-study and non-blind control arm a possibility to buy HT.

In the early 1990s, Estonia was still a maiden country for conducting a trial with HT. HT use did increase in the 1990s, but by 2000 it was still notably lower than in Finland [24]. In an Estonian survey in 1998, only 4% of women aged 45–64 reported current use of HT [25] compared to 34% in Finland in 2000 [26].

By the mid-1990s, prices and salaries in Estonia were lower than in most Western European countries, but the infrastructure (including health services) was good and western-style legislation and regulations had been developed [27]. The Estonian Health Insurance Fund Database, the Estonian Cancer Registry and the Estonian Mortality Database made it possible to collect information about women's health and health services use. The Estonian Health Insurance Fund Database is unique and includes information on all health care visits, diagnosis and prescriptions [28].

Various changes in legislation, relevant institutions, and financing occurred during our trial. The local co-ordinating centre, the Institute of Experimental and Clinical Medicine (EKMI), was also reorganized and it became the National Institute for Health Development (TAI) as of May 2003. Our study clinics were financed by the Estonian Sickness Insurance Fund (later Health Insurance Fund) [29]. Women's health outcomes were abstracted for the EPHT trial from the Health Insurance Fund register. Changes in the administration of the Health Insurance Fund required us to conduct several negotiations to ensure continuation of the trial. Women's recruitment visits were classified as health check-up visits and they were recorded in the Health Insurance Register.

During the trial some unexpected expenses occurred (increase in salaries, payment for physicians, reimbursement of mammograms for participants, etc) and prices increased much faster than could have been expected at the time of planning. During the trial period salaries rose about 30%, which tightened the trial budget, which had been planned in 1997. Originally only midwives were to be paid and physicians were to be compensated by commodities like international medical journal subscription or participation in international congresses. This turned out to be impractical and physicians received compensation per recruited woman.

While Estonia has good health registries, at the planning phase of the EPHT, Finland had better developed practices in data protection than Estonia and so these were adapted to our trial. During our trial, various changes were made to the Estonian data protection laws that led to reduced access to various registries. The 1996 Data Protection Law, or its updated 2003 version, were not clear in regard to the use of registries for research [30], with the ambiguity in interpretations causing additional work and delays in obtaining outcome data. A rapid turnover of personnel in the ministries reduced experience in data protection practices. The trial participants had signed informed consent permitting their survey and health examination data to be linked with health registries, but the completeness of registries became a problem because the data protection authority challenged the updating of these registers. Furthermore, maintaining up-to-date addresses for the participating women who had moved residence became difficult, because we were not allowed to check addresses from the population registry.

Data collection for disease outcomes in our trial was mainly based on registries, and new and changing regulations meant extra negotiations and time delays. Nevertheless, after difficult negotiations within the ministries all necessary data other than deaths had been obtained up to the end of 2004 as planned.

At the start of the recruitment in 1999, screening for breast cancer with mammography was not in use in Estonia, not even among HT users. If a woman had breast problems she was referred to a mammologist, a specialized physician. To compensate for the missing screening program we advised our study women to regularly palpate their breasts through the Mama breast self-examination program [31]. However, beginning in 2000, mammogram screening in Estonia was gradually introduced. This development – in addition to the advice given by local mammologists – led us to add mammogram screening for all trial women who had already been in the trial for two years (Figure 2). Those women who were eligible for local free of charge mammogram mass-screening programs were encouraged to use the service. For others the costs were covered by the trial, and they were unexpectedly high.

Many foreign drug companies were interested in Estonia and the number of approved clinical trials increased from five in 1992 to over 80 in 2004 [32]. With the increasing number of trials, we had to ensure that the women participating in our study were not recruited to other trials. This could effect EPHT results or reveal treatment medication to women in the blind sub-trial.

### Using a licensed regimen

The estrogen used in our study (CEE) had been available since the 1940s [33] and combined estrogen-progestin since the 1970s [34]. A wide variety of preparations have been available for climacteric women [35]. When we started our trial, HT was already available and in use in Estonia, including the specific trial regimen.

Studying an established therapy had its advantages and disadvantages. An advantage is that the ethical burden is lessened because women outside the study can be freely prescribed the drug. A disadvantage is that compliance in the non-treatment arm can be easily compromised through purchase of the drug outside the study. This was not a major issue in our trial: only some women receiving the placebo and less than 10% in the non-treatment arm had been subsequently prescribed HT by the exposure end [36].

The WHI researchers had chosen conjugated equine estrogens (CEE), which is the most widely prescribed preparation in the USA, but rarely used in Europe. WISDOM approached major HT manufacturers, but Wyeth was the only company prepared to supply drugs and matched placebos [10]. So, both the WHI and WISDOM ended up studying the same type of HT out of the dozens of preparations available.

As a consequence, most health data now available in relation to long-term HT use are based on one type of drug and, for example, transdermal preparations remain untested over the long-term. Before the start of the WHI and WISDOM, the applicability of specific HT preparations or formulations was not questioned [10], but later proponents of preventive HT have used the specific features of CEE as one argument to support continued use [19].

### Views of women and physicians

According to the EPHT pilot survey (n = 2 000, response rate 69%) 53% of the women were of the opinion that the climacteric is a normal phase in a woman's life which does not need medical treatment and only 17% disagreed [25]. Few women were familiar with HT, 11% had ever used HT and only 6% of the women supported HT for all postmenopausal women [25]. Women's inexperience and hesitation in regard to HT may have contributed to the low adherence in the placebo and HT-arms in the trial [36].

On the contrary, according to our survey in 2000, Estonian gynaecologists favoured HT and 37% recommended HT for postmenopausal women in climacteric [37]. GPs referred almost all of their patients with menopausal symptoms to a gynaecologist. Physicians thought that the increase in the use of HT in Estonia was more based on changes in physicians' opinions than that of women. Gynaecologists had frequently participated in education on HT, and education was often supported by industry [24]. Trials can be considered as a means to increase drug use, and this possibly contributed to physicians supporting our trial.

In preparing the information leaflets for women, it was revealed that still in the 1990s many physicians had paternalistic behaviours and that it was not considered crucial to inform patients. Discussions about cancer risks still seemed to be a taboo [24].

When we started our trial in Estonia, the culture of doing clinical trials was still new. Compared to the pharmaceutical companies, our resources were small, but due to the strong local academic participation, we had been successful in recruiting capable gynaecologists and midwives and had only a small turnover of research personnel. As the trial personnel had no prior epidemiological knowledge, the trial co-ordinators organized free of charge semi-annual seminars for the clinical staff on research methodology and controlled trials. In addition, when the local research assistant visited the clinics to follow the recruitment process and collect weekly summary sheets, she also discussed the trial progress with the midwives, and offered help in the case of problems. The trial staff and participating women could contact the trial co-ordinators any time by phone and by mail, and the midwives at trial clinics had special calling hours for trial participants. Trial participants were mailed a personal birthday greeting throughout the trial.

### Practical issues

An application to the Estonian drug control authority for permission was unproblematic. However, the first shipment of drugs was in bulk and the Estonian law required that the tablets have to be packed into vials and labelled by a pharmaceutical company. Because of the small number of tablets packing would have to be done by hand, and many negotiations were needed before we reached an agreement with a local pharmaceutical company. The next shipment of drugs was actually pre-packed, with 215 tablets in each vial i.e. for seven months use, but we still needed a pharmaceutical company to put the labels on the vials. The company who had done the packing was unwilling to do it and we had to find a new company. More importantly, this changed the time of the second and later visits: the six-month period between the visits was changed to seven months.

In the EPHT trial, all the trial clinics were located in Estonia, but the main scientific co-ordination was in Finland. Health care was different between the two countries. Personal contacts and open discussions were most valuable in bringing to light the different practices and in finding solutions. Many external changes placed additional demands especially on the local coordinator, who had to seek new solutions and contacts.

## Discussion and Conclusion

### Keys for success

Treatment in the EPHT trial was stopped earlier than planned, but the time was sufficient to provide answers to our short-term research questions. Taking into account all the outside changes occurring during the trial, we are satisfied with the trial process. However, the low adherence and the relatively short exposure time reduced the power of the study. A very good collaboration between the Trial Steering Committee, the Data Monitoring Committee, the trial coordinators, and the trial clinics made it possible to find a balance between the needs of achieving responses to the trial aims with a limited budget and simultaneously maintaining the safety of trial participants. Flexibility in finding the best solutions in every situation was the main key for success.

The relative success of the trial was due to keeping the trial staff and participants continuously motivated as well as tireless negotiations with authorities. Repeated changes in the health care system and in legislation were keenly followed up by appropriate actions (meetings with ministers, data protection authorities, and other stakeholders, articles in newspapers, discussions on radio). Preventive drug trials usually have a long duration and the pressures for changes to the protocol are strong. Our trial was a small-scale trial which meant that it was easier to manage in the face of such constantly changing circumstances: the organization was flexible, while participants both in the decision-making board and the clinics were fully committed to the trial. Rapid changes are currently possible in every society and co-ordinator must be constantly aware of the trial environment and identify possible threats which can affect the trial process and be prepared to act in case these changes occur.

Financing is a major challenge in a long-term preventive trial. In the Finnish financing system, funding decisions usually cover only a couple of years at a time, budgets are often made on current prices and resources are bound to budget years. More flexibility and longer commitment in financing would help in administering a long-term trial.

In preventive drug trials the costs of drugs are usually high. Even publicly funded researchers usually ask for drugs to be donated from drug companies. Drug companies may not be so enthusiastic about sponsoring trials of an established therapy, because it is a financial risk. Beneficial results may increase sales, but not necessarily of the specific product of the sponsoring company. If the results are negative, pharmaceutical competitors may attempt to deflect the impact by insisting the negative results apply only to the drug used in the study. In the case of the WHI and WISDOM, Wyeth Ayerst was the only pharmaceutical company willing to take the risk. In 2001 Wyeth covered 70% of the global market [38]. When the non-beneficial results from the WHI were released in June 2002 sales of HT in the USA declined, with the decline in Wyeth products being especially dramatic [39-41]. Those companies that did not take the risk of donating drugs to the trials could now argue that their regimens are different from Wyeth's and the trial results do not apply to their products.

An important question is who should prove the effectiveness of an (old) drug for a new preventive indication. Preventive drug trials are often long-term, and usually need large numbers of participants, thus increasing the costs involved [42,43]. Public funding is crucial in maintaining the independence of the trial from commercial biases. In the future, some procedure to share the costs of large trials should be negotiated where benefits and harms of licensed drugs need to be (re)evaluated. Public support for funding is needed for making evidence-based decisions in health care. Also, amendments to legislation for regulating the obligations of drug manufacturers to participate in post-marketing studies are needed.

A big threat to long-term trials is new information from other trials challenging the hypotheses and initial reasoning of the trial. In the case of WISDOM, a large-scale, well-prepared trial was terminated in an early phase. The wisdom of that decision can be questioned, especially in the light of the slow changes in the practice of HT use and the current criticism of a lack of information. Many questions about HT effects are still unanswered. However, there is very little to be made to this extraneous threat to long-term trials, besides reconsiderations of the norms used in terminating trials. The role of ethical committees and of data monitoring committees needs to be further specified on how to interpret information from other studies and make decisions about the pre-term stopping of trials.

## Competing interests

The authors declare that they have no competing interests.

## Authors' contributions

All authors have participated in designing the study. SLH is the coordinating investigator; PV is the coordinator in Estonia and EH is the director of the trial and MR is the director of the trial in Estonia. SLH drafted the manuscript and other authors commented and revised it. All authors read and approved the final manuscript.

## Pre-publication history

The pre-publication history for this paper can be accessed here:


